# Evaluation of blended medical education from lecturers’ and students’ viewpoint: a qualitative study in a developing country

**DOI:** 10.1186/s12909-020-02388-8

**Published:** 2020-11-30

**Authors:** Mohamad Jebraeily, Habibollah Pirnejad, Aram Feizi, Zahra Niazkhani

**Affiliations:** 1 Department of Health Information Technology, Urmia University of Medical Sciences, Urmia, Iran; 2 Patient Safety Research Center, Clinical Research Institute, Urmia University of Medical Sciences, Urmia, Iran; 3 Nephrology and Kidney Transplant Research Center, Clinical Research Institute, Urmia University of Medical Sciences, Urmia, Iran

**Keywords:** E-learning, Blended education, Virtual education, Qualitative study, SWOT analysis, Evaluation, Information technology

## Abstract

**Background:**

To improve the quality of education, many academic medical institutions are investing in the application of blended education to support new teaching and learning methods**.** To take necessary measures to implement the blended learning smoothly, and to achieve its goals, we aimed to identify its strengths, weaknesses, opportunities, and threats (SWOT) from its key users’ viewpoints.

**Methods:**

A qualitative study consisting of 24 interviews with lecturers and students and document analysis was conducted at Urmia University of Medical Sciences, in Iran, in 2018. The SWOT framework was used to analyze the data.

**Results:**

The most important strengths were the promotion of lecturer-student interactions, the focus on students’ learning needs and self-learning, and problem-solving skills. The supports of university executives, alignment with the national health education transformation plan, and access to the shared infrastructures of the national virtual medical science university were opportunities to facilitate its implementation. However, this endeavor had weaknesses such as bottlenecks in technical, organizational, and human resource infrastructures and lack of culture readiness. The threats envisioned for its maintenance were its dependency on the education transformation plan and the lack of an independent e-learning center for better planning and support services, lack of proper evaluation and supervision of virtual activities, and insufficiency of the privileges considered for users.

**Conclusions:**

One of the important implications of this study is that different aspects surrounding blended learning might work as a double-edge sword from time to time, which requires a thorough overview. While retaining the strengths and enjoying the opportunities in such interventions, the weaknesses should be recognized and threats are faced and addressed. Therefore, if the SWOT items are considered mindfully, they can help to adopt the right implementation strategies to reap full benefits.

## Background

To preserve its quality, medical education is facing serious challenges nowadays, such as the upward curve of growing medical knowledge, changes in health service delivery, and also changes in the learning interests of students in health and medical sciences [1]. In this context, traditional methods of medical education can no longer meet the needs and requirements of current medical students; and, therefore, cannot support them to achieve and maintain the notion of active and continuous learning [2, 3]. Novel and innovative teaching and learning methods are then required to improve student skills in critical thinking and academic achievements in medical education.

With the development of Information and Communication Technologies (ICTs), e-learning is getting the attention it deserves in the education of medical sciences. To improve the quality of education, many academic medical institutions are progressively investing in the application of e-learning and to support new teaching and learning approaches and strategies to help today’s learners to succeed [2, 4, 5]. Meanwhile, pure virtual education is not recommended in medical sciences, because it is increasingly emphasized that the virtual cannot replace the traditional face-to-face education and merely is complementary to it. Therefore, currently, one of the main approaches towards e-learning is the blended (hybrid) learning in which virtual education is combined with the traditional classroom-based education [6–8]., as an innovative component of blended education is in fact one of the most effective methods of computer-assisted learning by providing interactive educational content and managing the ongoing learning processes [4]. It enables the expansion of educational opportunities beyond class rooms, by providing better access to educational content while bypassing any time and space limitations [5, 9, 10]. It helps to stabilize the guidance role of university lecturers and to optimize the teaching and evaluation methods by promoting an independent, proactive, self-directive, and learner-centered education. However, despite many positive features, students, teachers, and institutions face challenges while using blended education, which need to be addressed, mindfully [11].

To achieve the goals of blended education, recognizing and addressing its challenges (especially of its online components) and adopting the right implementation strategies can guarantee its success [11, 12]. When intending to implement blended education, any organization should consider its strengths and weaknesses and try to recognize its facilitators and probable barriers and limitations to examine the readiness to its implementation. In this regard, evaluating the status of an organization in various dimensions such as its technical and human resource infrastructures is required, for example [9, 10, 12, 13]. To evaluate such issues while implementing an intervention, the strengths, weaknesses, opportunities, and threats (SWOT) analysis is a useful method [13, 14]. This method aims to recognize strengths and weaknesses in inner dimensions of an organization and opportunities and threats in outer dimensions.

The SWOT analysis can be performed to evaluate the implementation of e-learning based on the viewpoints and experiences of all stakeholders, especially university lecturers and students [15]. The result of research based on the SWOT analysis for the evaluation of blended education has shown that the most important strengths and opportunities include meeting the needs of students in the third millennium, creating equal opportunities for learning, promoting self-learning skills, facilitating access to the updated electronic contents, enabling inter-universities’ cooperation, and its flexibility and use of a variety of teaching and evaluation methods [13–20]. These research have also pointed out that the insufficient motivation for or even resistance of some lecturers to engage in, limitations in ICT and network infrastructures, lack of proper training or supporting services, lack of culture readiness in medical organizations and disrespecting the copyright protection law and intellectual property rights are the most important weaknesses and threats.

A systematic review published in 2015 by world health organization (WHO) examined the e-learning and blended learning methods and highlighted that many methodologically sound studies came only from developed countries; making it difficult for low- and middle- income countries to apply their findings in their low-resource contexts [21]. Recent studies have encouraged researchers to evaluate e-learning opportunities and report the challenges in low- and middle- income countries with special emphasis to focus on issues and features beyond individual learner perception in order to have a holistic view to inform and guide a strong e-learning system [21, 22]. Once in Iran, there was a dearth of activities and infrastructure in e-learning strategy in medical universities [23]. But, fortunately, since 2015, a wide range of activities has been carried out by implementing the education transformation and innovation plan in Iranian medical universities to promote the quality of medical education with special emphasis on the expansion of virtual education as a component of blended learning [24]. As one of the high-rank medical universities in the country, Urmia University of Medical Sciences (UUMS) as well invested in the expansion of e-learning for medical and health sciences students. To take necessary measures to implement the virtual component of blended education smoothly and achieve its goals, any organization needs studies to identify its strengths and opportunities as well as its challenges in terms of weaknesses and threats [25–27]. Thus, we aimed to evaluate the virtual component of blended medical education from the perspectives of its key users using the SWOT analysis, aiming to get insight into the organization’s situation in inner and outer dimensions.

## Methods

This study is a qualitative research conducted at the UUMS in 2018 using semi-structured interviews and the documents associated with the development and implementation of the blended learning available at the university and the deputy of education at the Iranian ministry of health and medical education. The participants in our interviews consisted of 11 faculty members and 13 masters’ and professional doctorate students at the UUMS that had used the blended education in more than four university courses during at least two educational semesters in 2017–2018. These participants were purposefully chosen because they had valuable information and gained sufficient experience on this topic at the time of our study. Our faculty members used a commercial learning management system (LMS) that was provided by the ministry of health and medical education to support the implementation of its education transformation and innovation plan. The common format of teaching in the university was face-to-face classroom teaching at the time of this study. The lecturers interested in blended learning could deliver the online component of their teaching hours up to four out of 17 total hours per one university credit during a term. These virtual teaching sessions supplemented the classroom teaching either by expanding on the subjects already taught in the classrooms or by covering new subjects. However, they could only cover the theoretical and procedural trainings, and practical skills had to be delivered by face-to-face training.

After selecting the participants, appointments were held at a time and place most convenient for the participants. Participation in the study was voluntary and all participants gave verbal informed consent to participate. The interviews were performed one-on-one and face-to-face. The interviews lasted 35 to 50 min. After gaining permission, the interviews were audio-recorded with the consent of the participants after ensuring that the confidentiality of information provided by them would strictly be preserved. The data was not encrypted but they stored in a secure place to preserve security. The study aims, importance, and necessity were first explained at the beginning of each interview. Then, four main questions were asked followed by supplementary questions about each to encourage further discussion about the participants’ perceptions and experiences on the subject. It is worth mentioning that these four main questions were: 1) what are the strengths of the virtual component of blended education you used? 2) what are its weaknesses?, 3) what opportunities do you think this can be created for education in our context?, and 4) what threats do you think it can pose to the education? After each interview, the recorded information was checked few times before conducting the following interviews. The interviews were transcribed verbatim. We continued our interview participant recruitments till our analysis showed that data saturation was achieved. Thematic analysis was used to analyze the qualitative data. Content analysis of the interview transcripts and also the documents was performed on the basis of the dimensions provided by the SWOT framework. In this method, after careful reading of the interview transcripts by the researchers, the main codes related to the strengths, weaknesses, opportunities, and threats were recognized and then the themes were analyzed in a systematic way. To improve the credibility of our data and analysis, interview transcripts were sent to and checked by the interviewees in written form. Any text correction by the participants in this phase was used in the final analysis.

## Results

Eleven (six females and five males) faculty members with an average age of 39.5 years old and 8.6 years of working experience participated in our study. They were members of different faculties of medicine, dentistry, pharmacy, health sciences, paramedical sciences, and nursing. They contributed in the teaching different subjects including parasitology, anatomy, radiology, internal medicine, dentistry, pharmacology, occupational health, medical entomology, and medical informatics. Our 13 student participants (eight females and five males) were seven masters’ (including medical informatics, nursing education, parasitology, anatomy, and physiology) and five doctoral students in medicine, pharmacy, and dentistry with an average age of 21.7 years. These students had used blended education between 2017 and 2018.

Content analysis to evaluate the blended education in our context from the lecturers and students’ viewpoints based on the SWOT framework revealed 30 initial codes in four major themes (Table 1) as followings.

**Table 1:** Findings according to the SWOT framework

**Strengths** • Focus on students’ learning needs • Matching with students’ interests in digital tools • Flexibility • Variety of teaching and evaluation methods • Improving productive lecturer-student interactions • Improving self-learning and problem-solving skills	**Weaknesses** • Lack of training courses • Deficiency in IT and network infrastructures and human resources • Lack of institutionalization of virtual education culture • Lack of an independent LMS • Complex and time-consuming task of virtual education • Lack of sufficient suitable feedback • Lack of information and reminder • Lack of required infrastructure and skillful human resources
**Opportunities** • Health education transformation and innovation plan of the ministry of health and medical education • The university’s and ministry’s senior executives support • Foundation of virtual university of medical sciences • User-friendly and easy-to-use LMS to be acquainted with • Access to the updated and standard electronic contents • Development of regional and international cooperation	**Threats** • Dependence of virtual education development on the national education transformation plan • Resistance of the lecturers to use the virtual education • Lack of proper evaluation of virtual education • Non-compliance with the principles of copyright and intellectual property • Insufficiency of the privileges considered at the university for people who use the virtual education • Lack of an independent virtual education center at the university

### Strengths

According to the participants in this study, the following issues were the main strengths of using blended education in the UUMS:

#### The focus on the learning needs of students

Our study participants believed that the virtual component enabled the faculty members to better understand students’ needs in learning through close and constant interaction with them; and, therefore, the faculty members could be able to tailor the education according to their students’ needs and wishes. The following quotes clearly show this point:

*“By asking from the students and the feedback that they provide me with [through the LMS], I can understand their learning needs more and I can try to include the materials that must be learned by the students in the LMS”. (Faculty member 5)*

*“If I have a question in my mind after studying the educational contents and sources, I can easily ask the lecturer in the LMS”. (Student 2)*

#### Matching students’ interests in and desire for using digital tools

According to the interviewees, the popularity of digital and IT tools among university students provided the potential for the development of blended education.

*"Students are interested in learning via multimedia tools and the virtual education provides them with this chance." (Faculty 5)*

*"I like to learn the material using my cell phone or laptop, as it is pleasant for me and always available." (Student 5)*

#### The flexibility of the virtual component of blended education

Access to the LMS without any time and space limitations and its various capabilities had given better teaching opportunities for lecturers and unique self-directed learning chances for students.

*“[ … while using e-learning module] whenever I find useful material, I can upload it into the LMS. I can answer my students’ questions and also check their homework in my free time”. (Faculty 1)*

*“I can have access to the learning contents multiple times at any time and location that I wish and I can learn more in this way”. (Student 3)*

#### Opportunity to use a variety of teaching and evaluation methods

The use of electronic content production software enabled an interactive content presentation mode. It allowed the creation of various kinds of exams using multiple evaluation methods, as well.

*“By taking the advantage of available capabilities in the virtual education such as [uploading] multimedia contents and designing homework, exams and [other kinds of] interactions, teaching and evaluating methods are improved”. (Faculty 7)*

*“If the electronic content has a good quality and the lecturers check exams and the answers to the homework in the LMS and provide feedbacks, the virtual education can become much better than the traditional methods”. (Student 5)*

#### Encouraging students to take part further in the learning process

The virtual education could encourage the learners to take part more actively in the learning process. This was achieved by creating an environment where university students can compete and cooperate healthily.

*“The capabilities available in the LMS such as homework delivery and forum management motivate students to study more and to increase their chances for further cooperation in learning”. (Faculty 10)*

*“To get involved in the group discussions and deliver my homework, I have to study more to have better participation [record] and to keep up with my classmates”. (Student 12)*

#### Improving lecturer-student interaction

Access to the lecturers and also their supervision on students’ learning processes were improved in blended education.

*“By leaving messages to inform students, I have more access to them. Also by checking the LMS reports, I can have better supervision over their progress”. Faculty 4*

*“I can have better access to the lecturer in the LMS and I can easily ask my questions or provide [him/her] with my suggestions”. (Student 9)*

#### Improving self-learning and problem-solving skills

The study participants believed that the role of the lecturers was as facilitators in the virtual component and the students could develop self-learning and problem-solving skills.

*"In virtual education, the role of the lecturer is mostly introducing suitable resources, developing problem-based homework, and giving feedback. Thus, the student has to learn him/herself and should be able to practice the problem-solving skill." (Faculty 11)*

*"By studying the resources [references] and doing homework and [solving] exams, my self-confidence is improved in learning the new materials and solving problems and I feel that I can myself learn many things and I [can] just ask for guidance from the lecturer whenever it is needed. (Student 6)*

### Weaknesses

The following points were mentioned as the weaknesses of the virtual component in our context, deserving greater consideration for improved efficiency.

#### Lack of training courses for students and staff on the virtual component of blended education

To use the full potentials of the virtual component, people needed to learn the required skills to work with and design and develop electronic contents.

*"Sufficient training courses are not held at the university and I feel I do not have the required skills and readiness to use the virtual education. (Faculty 3)*

*"We did not have suitable training to use all the capabilities in the LMS; and sometimes, it has really caused confusion. (Student 13)*

#### Weak support and troubleshooting services

The participants in the study stressed on the issue that professional consultations should be offered to the users to start using the virtual component and the focus should be on the meeting of their current demands and also new needs in the LMS.

*"There is no one who can provide the required guidance for designing the teaching scenarios and producing electronic content and our new needs in the LMS are rarely cared for. (Faculty 2)*

*“When we have questions or face problems in the LMS, there is no proper support system”. (Student 7)*

#### Deficiencies in information technology (IT) and network infrastructures

Low internet speed and its frequent disconnection had created problems in using the virtual component making the lecturers and students disappointed.

*"Limitation in uploading the materials sometimes makes me unable to upload [my] electronic contents into the LMS. The problems and disconnections of the internet at the university [also] waste a lot of [my] time. (Faculty 6)*

*"We have lots of internet disconnections in the dormitory and the low internet speed makes me log into the LMS several times before I can access it. (Student 11)*

#### Lack of institutionalization of the blended education culture at the university

The participants of this project thought that virtual component of blended education had not yet been understood as a part of the medical education system at the university. It was usually considered as an independent and voluntary education system; therefore, all the lecturers and the students were not commonly aware of its capacities and necessity.

"*Most of the colleagues do not entirely aware of the goals and advantages of virtual education and the necessary information has not been provided [to them] in this regard. (Faculty 5)*

*"Only some of the lecturers have held their classes in a virtual way and only for few sessions. Still, most of the lecturers and students do not have enough information and even a desire to use the virtual education. (Student 10)*

#### Deficiency of skilled human resources to support the

At the time of this study, the number of experts to provide professional guidance and consultations about the virtual component seemed to be very limited impacting the optimal e-learning implementation.

*"The number of e-learning specialists and technology experts in education at the university is very limited and [then] sufficient support for producing electronic contents is not provided. (Faculty 9)*

#### Lack of an independent LMS at the university

Because the university was dependent on the LMS of the ministry of health and medical education, some problems were encountered and limitations were experienced, as echoed in the following quote.

*"As our university does not have an independent LMS, then our needs and demands are not considered in it. Even, there is no system administrator from our university; and thus, sometimes solving the existent errors and problems [in the LMS] becomes very time-consuming." (Faculty 1)*

#### Complex and time-consuming design and implementation of the

The participants stated that utilizing the virtual component of blended education was somehow complex and difficult at first, which required learning some skills and spending lots of time.

*"Producing suitable electronic contents is complex. [Also] Evaluating homework and providing suitable feedback to students need lots of time. (Faculty 3)*

*"Doing some of the homework is time-consuming and challenging; and sometimes, we don’t understand what the lecturer means, and then we have to text him [to ask him] to explain it a little bit more." (Student 3)*

#### Lack of sufficient and suitable feedbacks on the assignments by the lecturers

Sometimes, lecturers had fewer interactions in the LMS for different reasons.

*"The lecturers do not provide proper feedback for us to understand how our learning process has been and they don’t take part in the conversations, seriously. (Student 2)*

#### Lack of information and reminder

Due to the lack of any reminder/alert in the LMS, it was suggested that an on-time reminder/alert about the last activities in it should be available and sent via email and text.

*"The LMS is not able to [send] text [messages] the latest activities done by the lecturers or students in the LMS and it cannot remind the assignment and exam timings." (Faculty 9)*

*"When the lecturers upload some assignments or contents in the LMS, we get to know it very late. If there were some possibilities of letting us know by email or text messages, it would have been much better, then. (Student 11)*

### Opportunities

#### Implementation of the national transformation and innovation education plan

The support provided by the ministry of health in the form of a “virtual education package” was an opportunity to expand the blended education in the study environment as it is obvious in the following quote:

*"The existence of virtual education package in the transformation plan has led to the formation of a working group with the presence of senior managers and skillful and interested people and [since then] good progress has been achieved in developing the virtual education.’ (Faculty 2)*

#### Support of the ministry’s and the university’s senior executives

The transformation plan had urged the senior managers at the university to support this endeavor and to provide the necessary infrastructure as much as possible.

*"Monitoring of the virtual education at the university by the ministry has given greater motivation to the senior executives and their support." (Faculty 3)*

#### User-friendly and easy-to-use LMS to be acquainted with

Both lecturers and students referred to the LMS as one of the success factors of the implementation of blended education in our context mainly because they found it very suitable and user-friendly.

*"The LMS has provided a proper environment for the virtual education activities and it is easy to use and it graphically looks good, as well. “Faculty 10”*

*" … the work environment has been designed well in the LMS and it is easy to work with it and I feel it is helpful. (Student 4)*

#### Foundation of a virtual university of medical sciences in the country

A national virtual medical science university, with all necessary infrastructures, had recently been founded, which offered standard electronic contents and training courses even for instructors. This event had in a great deal helped to improve the blended education in other universities.

*"The virtual university has played an important role in developing virtual education in other universities by offering necessary facilities such as free access to the LMS and providing relevant training courses." (Faculty 3)*

*"When the lecturer introduced the virtual medical science university’s website, I got lots of useful information about the advantages and capacities of the and also some information about how to work with the LMS and got interested to take part in the courses." (Student 11)*

#### Development of regional and international cooperation

Blended learning provided the opportunity to use the available potentials in the national and international universities to hold common courses at the regional and international levels in a virtual way.

*"The virtual education creates lots of opportunities for cooperation between universities to hold joint courses." (Faculty 9)*

### Threats

#### Full dependence on the national transformation and innovation education plan

At the study time, the development of blended education was largely due to the obligation imposed by the ministry. Participants were highly concerned about its future in a case for any reason the national plan would stop.

*"If the transformation plan stops and the supports and follow-ups become less intense, the development of the virtual education will face lots of problems due to the lack of infrastructures and budgets." (Faculty 3)*

#### The resistance of the lecturers to use the virtual component of blended education

The development of virtual education required learning of new skills and also changing the traditional methods of teaching and evaluation of students, which perceived difficult by some of the lecturers.

*"Some of the lecturers are reluctant to change their teaching methods, which needs acquiring new skills to use the virtual education, and [that’s why] they are resistant to do it." (Faculty 8)*

*"When we ask some of the instructors to use virtual education in their teaching [methods], they reject it." (Student 7)*

#### Lack of proper evaluation of the virtual education associated activities

At the time of the study, certain standards had not yet been available in the virtual education scenarios and electronic contents; and then, its evaluations were not complete, clear and, precise.

*"The current evaluation [of the virtual education at the university] is so general. The lecturers’ and students’ activities in the LMS are monitored less often and no feedback is provided." (Faculty 7)*

*"Students’ satisfaction about the quality of virtual education and its influence on the quality of their learning are not assessed well." (Student 6)*

#### Non-compliance with the principles of copyright and intellectual property of electronic contents

The interviewees emphasized that if the rules of intellectual ownership of the electronic contents were not met or copyright laws not followed while using others’ contents, this would lead to problems for sure.

*"Unfortunately, some of the colleagues and even students do not follow the copyright laws and the exact reference is not provided for the contents used." (Faculty 10)*

#### Insufficient technical and non-technical infrastructures

Lack of technical infrastructures and the human resource and support services could create lots of problems while implementing and maintaining the sustained use of the virtual component.

*"The lack of an independent LMS and an acoustic room, and a shortage of e-learning and medical education specialists can lead to many flaws in the development of virtual education and cause frustration for faculty and students." (Faculty 6)*

#### Lack of advantages and privileges for those who use

Our interviewees believed that if necessary privileges and motivation are not provided for using blended education, its use will face obstacles.

*"Privileges for virtual education should be considered [for example] for promotion of the faculty members. Otherwise, they won’t invest enough time and effort to develop it and they will not be willing to use blended education." (Faculty 6)*

*"Some lecturers do not consider any special credit for our active cooperation in the virtual education, or they do not provide proper feedback and this makes us use the virtual education less often." (Student 6)*

#### Lack of an independent e-learning center at the university

The existence of an e-learning center to provide supportive infrastructures and services for blended education and also to evaluate the activities was perceived to be essential and vital for its development.

*"The development of virtual education at the university will not be comprehensive unless an independent e-learning center is considered, and a specific budget is allocated to it, and dedicated and specialized staffs are recruited to work with". (Faculty 2)*

## Discussion

The results of our study showed that the most important strengths of the blended education included the promotion of lecturer-student interactions and its flexibility and focus on students’ learning needs, impact on improving self-learning and problem-solving skills, and match with students’ interests in digital media. In our context, having the support of top university executives, its alignment with the health education transformation plan, and access to the shared infrastructure of the national virtual medical university were opportunities that facilitated the implementation of the blended education to a great deal. However, this endeavor had weaknesses such as insufficient technical, organizational, and human resource infrastructures at the university for students and lecturers, and lack of institutionalization of the blended learning culture. Moreover, it has been threatened by its total dependence on the national health education transformation plan, lack of an independent e-learning education center for better planning and support services, lack of proper supervision and evaluation of activities in the virtual component, and insufficiency of the advantages and privileges considered for the individuals who use the blended education. This partly explained the resistance to start using it, especially by lecturers.

Blended education can generally make changes in the ways that teaching, learning, and interactions between lecturers and students occur. Similar to others [28], our participants believed that this kind of education corresponds to the learning needs of students and can enable productive variation in teaching and evaluating methods. It can also promote lecturer-student interactions and motivate students to engage in the learner-centered education according to their experiences and backgrounds. Blended learning also bears the beneficial properties including the comprehensiveness of learning, its flexibility, emphasis on a learner-centered rather than a teacher-centered system, and exploiting modern and novel virtual methods [7]. Jefferson and Arnold [26] also stated that virtual education can provide an opportunity for mutual productive interactions between students and lecturers and the formation of training working groups. Meanwhile, the ready availability of affordable infrastructures for blended education can be one of the strengths to facilitate the migration from the traditional teaching model to this modern one in any educational environment. The results of a study in Libya [29], using the SWOT framework, showed that the accessibility of electronic contents for teachers and students to support e-learning was one of the strengths of higher education institutions. In our study as well, we found that the easy access to the LMS was displayed as strength. Besides to the above-mentioned issues, others have reported further benefits including matching the characteristics of the third-generation students such as their interest in digital tools and media, providing the opportunity for group learning and interaction between students, time savings and cost-effectiveness by not traveling to a college, and benefits of studying and working at the same time especially for postgraduate students, which all are in essence consistent with the findings of the current study [15, 25, 26].

Despite the strengths, the weaknesses of educational interventions can influence their effectiveness and maintenance. Issues related to the technical bottlenecks (e.g., associated to infrastructure and network), administrative affairs (e.g., limited availability of skillful e-learning human resources and insufficient training courses), personal factors (e.g., time-consuming production of virtual content and the lack of required commitment by the lecturers to give sufficient feedback on student activities) and socio-cultural issues (e.g., less developed e-learning culture) were found as weaknesses of the blended education. From the literature, it becomes clear that such issues are not specific to our study context. The lack of sufficient ICT infrastructures and suitable access to the internet are common barriers [11, 29, 30]. Another important weakness is the mismanagement of e-learning efforts and the underdevelopment of centers in distant areas, where they need the most [29]. A study in Romania using the SWOT analysis found that inadequate compatibility between the technical design of services and the psychological elements of the learning processes, inadequate supervision of student activities and their superficial learning, an imbalance between educational activities and ICT skills while using diverse educational methods and tools, and reduced communication between students and lecturers were among the shortcomings of the utilized e-learning [15]. In our study, students demanded the provision of appropriate, timely, and consistent feedback on their activities in the virtual component of education, because they perceived it as one of the shortcomings. If such feedbacks are addressed adequately, this can be turned into strong motivational support for students to engage in blended learning [30].

It is noteworthy that the blended learning initiatives should thoroughly be considered in their own educational context, and the purported benefits and strengths should not be taken for granted to hold true in different context. To put in other words, the implemented features of blended education and its management strategy can define its bottlenecks in an educational contexts in terms of for example access to lecturers, opportunities for training compared to that of in-person training, and appropriateness of the contents and materials for students, which might sometimes be in contrast to its widely portrayed strengths and opportunities [25]. Other gaps with the blended education mentioned in the literature are inadequate adaptations in the laws and culture and unsuitability of globally available contents for use in different cultural contexts and languages; even the accent and terminology used in virtual contents can challenge their effectiveness in education, too [31–33]. Regardless of these barriers, the crucial point is the support of governments, policymakers, and university executives, which can somehow help to address or resolve such existing shortcomings, for example, problems with technical and human resources or the provision of high quality, culturally appropriate and tailor-made contents.

There are facilitators for the adoption of blended learning in medical institutions that should be valued. A study at a Nigerian university modeled students’ intentions to adopt e-learning and found that factors such as attitudes towards e-learning, willingness to learn through e-learning, access to facilities, its mandatory use, and its usefulness can potentially affect the intention of users for adoption [34]. From the point of view in the present study, senior executives’ support in the context of implementing the health education transformation plan and the blended education’s correspondence with the interests of the new student generation in digital media has provided a great opportunity for its development and adoption. Kenan et al. [29] have indicated that gaining language and ICT literacy and skills with this innovative, time economic and convenient education are just some of the opportunities that the e-learning opens up for students. In another study, the opportunities that e-learning itself could create from the perspective of its stakeholders included creating a fundamental transformation in all aspects of education, from process to infrastructure, from teaching to evaluation, from teachers to students while the interest in education is increased with relatively low costs [15]. Noteworthy, the opportunities that e-learning opens up with lower costs and higher quality can be more accessible to low-income people [14, 35]. To tap such potentials, national education systems should invest in and move resources to revolutionize education, which can become handy especially in the time of a public crisis and emergencies such as disease outbreaks and air pollution.

Blended education is considered a preferred model of education in many universities, especially medical sciences, where the effects of many drawbacks in traditional education are mitigated by its capabilities to meet the various and multifaceted needs of university students. However, many challenges in education contexts can threaten to realize these potential capabilities. At our university, the full dependence of the blended education initiative on the national health education transformation plan, lack of an independent virtual education center, insufficiency in infrastructures, and failing to consider motivating privileges for those who use it was found to challenge the adoption. Following the coronavirus disease-19 (COVID-19) outbreak, some of these threats were the source of frustration when the university shifted from blended education to a completely online, virtual education in the majority of its subjects. In this regard, the dependency on an outside provisional infrastructure limited lecturers in uploading their content in terms of type and size or also made them face many downs in the LMS system due to the maintenance tasks of the national virtual university due to increased demand on their services. Other threats have also been mentioned in the literature such as a lack of employment for e-learning graduates [29, 31, 36]. Moreover, issues such as disproportionately exaggerating the positive roles of e-learning, the high cost of its implementation and maintenance, inadequate incentives to engage both teachers and students, and inadequate regulation of virtual teaching and learning activities can undermine its life [14, 15]. Therefore, to mitigate or compensate for the effects of such threats and challenges, policymakers should adopt strategies to manage it mindfully such as understanding and valuing internal and external effective factors by involving all key users in the decision and implementation of such interventions, clarifying the expectations from such educations, fully supporting e-learning initiatives, and allocating the required resources.

It is more than a decade, especially in higher education, that it is believed integrating teaching, learning, and technology is “a mandate, and not an option, anymore” [37, 38]. However, this had not been realized ubiquitously, until most recently (partly due to several common challenges faced by many medical institutions) [11]. Then, the pandemic of COVID-19 has profoundly changed the face of medical education and mandated the adoption of workable solutions such as e-learning at all levels to ensure the best outcomes and better preparedness for the future [39]. When physical distancing was required, in our setting all in-person classes transitioned to remote teaching via the LMS and other online videoconferencing platforms particularly for both theoretical and procedural courses and also group discussions and morning rounds for residents and students at the clinical placement stage. Although our study was conducted almost two years before the COVID-19 outbreak, the blended learning had been gradually strengthened its roots during this time. Drawing on this experience, our university could resume many of its educational activities within the first month after the outbreak.

## Conclusions

The identified strengths, weaknesses, opportunities, and threats in this study provide insights for policymakers and university executives regarding the management strategies for a smooth transition towards blended education and its adoption in order to reap the full benefits. At our university, the result was the further promotion of blended learning, which was very handy when the university had to switch from the blended education to completely on-line virtual education in the time of the COVID-19 crisis.

One of the most important implications of our findings is that different aspects surrounding innovations such as blended learning might work as a double-edge sword from time to time, which needs having a thorough overview. While retaining the strengths of an educational intervention and enjoying its opportunities, the weaknesses should be recognized and threats are faced and addressed. In our case, although participants reported that the productive lecturer-student interactions were improved with the virtual component, students yet questioned the lack of sufficient and on-time feedback from the lecturers on their activities. This implies that different types of interactions should still be monitored and promoted through online discussions, on-time feedbacks, and forums to compensate for the lack of rich face-to-face interactions that take place for clarifications or confirmations in classroom teaching. Another relevant instance is that, although, the availability of the LMS of the national virtual university was an opportunity to initiate the blended learning in our setting, the total dependency on an outside infrastructure limited lecturers in uploading their content in terms of size and types or made them face some interruptions in activities later on when the demand was high. Therefore, if the SWOT items are recognized and considered mindfully in each context, they can help to adopt the right implementation and management strategies to achieve sustainable benefits.

## Data Availability

The raw dataset on which the study is based on is available upon reasonable request.

## Abbreviations

ICT: Information and Communication Technologies
SWOT: Strengths, weaknesses, opportunities and threats
IT: Information Technology
LMS: Learning Management System
UUMS: Urmia University of Medical Sciences
COVID-19: Corona Virus Disease-19
